# Near-Complete Supraglottic Transection of the Larynx after a Motorbike Accident

**DOI:** 10.1155/2013/827902

**Published:** 2013-05-13

**Authors:** Sang Hwang, Samuel McGinness, Sim Choroomi, Ian Jacobson

**Affiliations:** ^1^Department of Otolaryngology and Head and Neck Surgery, Prince of Wales Hospital, Randwick, NSW 2031, Australia; ^2^Prince of Wales Hospital Clinical School, University of New South Wales, Randwick, NSW 2031, Australia

## Abstract

Severe laryngeal trauma is rare in the civilian environment and requires appropriate and timely surgical intervention. We report a case from Sydney, Australia, which was managed with open reduction and internal fixation of the larynx with resorbable plates. The use of resorbable plates for operative fixation of the larynx has rarely been reported in literature but may be a viable alternative.

## 1. Introduction

Laryngeal trauma is a rare injury in the civilian environment but can cause significant mortality and morbidity without timely, appropriate management. We report a case of a near-complete supraglottic transection of the larynx after a motorbike accident and discuss its surgical management.

## 2. Case History

A 26-year-old male presented to a tertiary referral hospital in Sydney, Australia, following a motorbike accident, during which the patient suffered an impact to the anterior neck from the upper horizontal pillar of an opened car door. On arrival, the patient was able to ventilate through the exposed lumen with minimal respiratory distress, and soft oral phonation was achieved by covering the wound.

**Figure 1:**
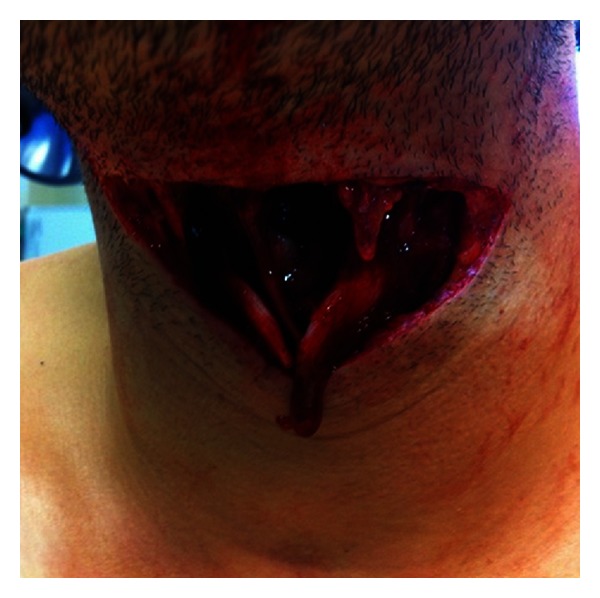
The laryngeal injury which was obvious at the time of initial presentation is demonstrated.

Initial assessment revealed a significant transverse penetrating injury with near-complete transection of the larynx between the hyoid bone and thyroid cartilage (Figure 1), with the cranial border of the thyroid cartilage evident protruding into the base of the wound. The great vessels of the neck were uninjured.

**Figure 2:**
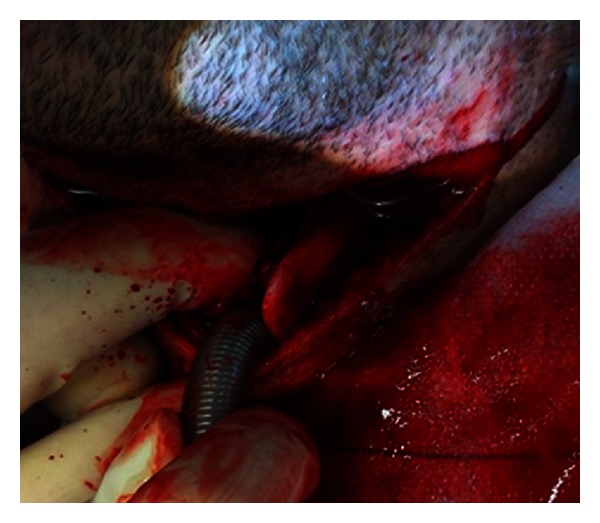
The patient was initially intubated via the open wound.

Decision was made to immediately proceed to theatre to secure the airway. Rapid sequence induction was initiated, and the patient was intubated using a reinforced size 8 endotracheal tube inserted via the open wound (Figure 2). The patient was then reintubated orally.

Intraoperative examination revealed an oblique, comminuted fracture of the anterior thyroid cartilage extending from the laryngeal incisure to the lower left margin, with muscular and mucosal transection through infrahyoid muscles. The median thyrohyoid ligament and thyrohyoid membrane were divided with extension to the posterior laryngeal wall, corresponding to a Schaefer-Fuhrman type IV laryngeal injury. The anterior commissure of the glottis was separated in the midline. There was complete avulsion of the thyroepiglottic ligament at the petiole. 

Tracheostomy was first performed through a separate caudal incision. The comminuted nature of the laryngeal fracture required fixation with a resorbable tripolymer plate (Delta II, Stryker Corporation), which was secured with monofilament sutures (PDS, Ethicon), as the supplied screws were unable to be fixed to the nonossified thyroid cartilage. Considerable attention was paid to restore the cartilage to anatomical position to prevent foreshortening of the left vocal cord. The wound was closed with absorbable braided sutures (Vicryl, Ethicon) with particular attention to meticulous repair of the anterior commissure and laryngeal mucosa to prevent exposed cartilage. 

The patient was transferred to the ICU, where ventilatory support was rapidly weaned, and the patient was decannulated on day 9. Modified barium swallow (MBS) revealed moderate to severe aspiration of contrast. Formal nasoendoscopic assessment of swallowing was performed on postoperative day 12 showing mild aspiration of thin fluids only. 

**Figure 3:**
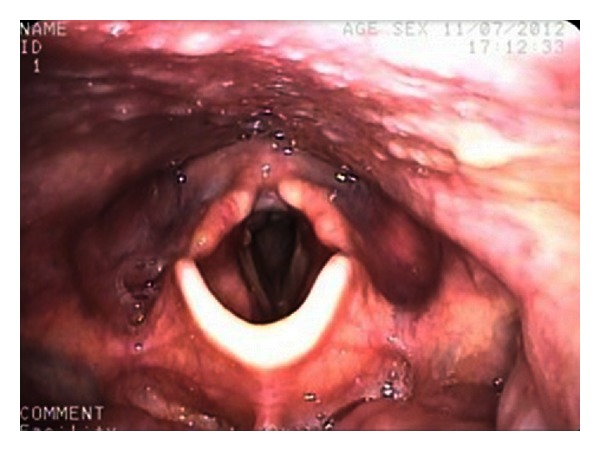
Nasoendoscopy at 5 weeks showed normal supraglottis, epiglottis, and vocal cord function.

The patient was discharged on day 14 tolerating a full diet and thickened fluids. A repeat MBS performed five weeks after injury revealed no aspiration of food or fluids. Nasoendoscopy revealed a normal appearance of supraglottis and glottis with preservation of full vocal cord function (Figure 3). Twelve months after injury the patient exhibited a normal voice to the trained and untrained ear, with full swallowing function and a normal airway.

## 3. Discussion

Penetrating laryngeal trauma resulting in laryngeal fracture and mucosal disruption is a rare event accounting for less than 1% of all cases seen in major trauma referral centres [1]. It occurs more frequently in military settings, especially in the context of shrapnel injury [2].

Early management of laryngeal trauma is crucial for a satisfactory outcome with regards to a patient's airway, phonation and swallowing [3, 4]. In a retrospective study of 564 patients, tracheostomy performed within 24 hours of presentation was associated with a shorter period of ventilator dependency, ICU admission, and overall hospital stay when compared to those established after 24 hours [4]. 

Several techniques to reconstruct the larynx are reported, with the key principle of anatomic reduction and internal fixation of laryngeal fracture and meticulous soft tissue repair being vital to a satisfactory outcome [1, 5]. The use of sutures alone or with metal adaptation plate fixation devices for this purpose has been documented by a number of authors [5–7], but aside from small case series, there is no significant data on the use of resorbable plates for laryngeal fracture [8, 9].

Resorbable plates, more commonly used in craniofacial fractures, have the theoretical advantage of avoiding long-term complications of metal plates such as plate migration or extrusion, interfering with diagnostic radiology and intraoperative pliability [8]. However, their routine use in managing laryngeal fractures has not yet been established as standard practice. Following our experience with resorbable plates in this patient, we believe these devices are a viable alternative for fixation of laryngeal fractures, and further research is required to validate its use. 

With early surgical intervention and meticulous anatomical reconstruction, postoperative outcomes are generally favourable, with several retrospective case series reporting minimal long-term morbidity in airway, phonation, and swallowing outcomes [1, 3, 10]. In this patient, the meticulous reconstruction of the individual layers of the larynx including the mucosal surface, cartilage, and associated ligaments maximised the chances of achieving an excellent outcome.
